# Tumor-associated macrophages in oral premalignant lesions coexpress CD163 and STAT1 in a Th1-dominated microenvironment

**DOI:** 10.1186/s12885-015-1587-0

**Published:** 2015-08-05

**Authors:** Kazumasa Mori, Shigeki Haraguchi, Miki Hiori, Jun Shimada, Yoshihiro Ohmori

**Affiliations:** 1 Division of Oral and Maxillofacial Surgery, Department of Diagnosis and Therapeutics Sciences, Meikai University of School of Dentistry, 1-1 Keyakidai, Sakado, Saitama 350-0283 Japan; 2 Division of Microbiology and Immunology, Department of Oral Biology and Tissue Engineering, Meikai University of School of Dentistry, 1-1 Keyakidai, Sakado, Saitama 350-0283 Japan

## Abstract

**Background:**

Tumor-associated macrophages (TAMs) are implicated in the growth, invasion and metastasis of various solid tumors. However, the phenotype of TAMs in premalignant lesions of solid tumors has not been clarified. In the present study, we identify the phenotype of TAMs in leukoplakia, an oral premalignant lesion, by immunohistochemical analysis and investigate the involvement of infiltrated T cells that participate in the polarization of TAMs.

**Methods:**

The subjects included 30 patients with oral leukoplakia and 10 individuals with normal mucosa. Hematoxylin and eosin slides were examined for the histological grades, and immunohistochemical analysis was carried out using antibodies against CD68 (pan-MΦ), CD80 (M1 MΦ), CD163 (M2 MΦ), CD4 (helper T cells: Th), CD8 (cytotoxic T cells), CXCR3, CCR5 (Th1), CCR4 (Th2), signal transducer and activator of transcription (STAT1), phosphorylated STAT1 (pSTAT1) and chemokine CXCL9. The differences in the numbers of positively stained cells among the different histological grades were tested for statistical significance using the Kruskal-Wallis test. Correlations between different types of immune cells were determined using Spearman’s rank analysis.

**Results:**

An increase in the rate of CD163^+^ TAM infiltration was observed in mild and moderate epithelial dysplasia, which positively correlated with the rate of intraepithelial CD4^+^ Th cell infiltration. Although CCR4^+^ cells rarely infiltrated, CXCR3^+^ and CCR5^+^ cells were observed in these lesions. Cells positive for STAT1 and chemokine CXCL9, interferon- (IFN)-induced gene products, and pSTAT1 were also observed in the same lesions. Double immunofluorescence staining demonstrated that the cells that were positive for CD163 were also positive for STAT1.

**Conclusions:**

CD163^+^ TAMs in oral premalignant lesions coexpress CD163 and STAT1, suggesting that the TAMs in oral premalignant lesions possess an M1 phenotype in a Th1-dominated micromilieu.

## Background

Oral squamous cell carcinoma (OSCC), which accounts for approximately 2 % of total new cancer cases, is the most common type of oral cancer [1]. Despite recent advances in our understanding and in the treatment of other types of cancer, the five-year survival rate after diagnosis of OSCC remains low at approximately 50–60 % [2]. The survival rate of patients with early-stage OSCC is higher than that of advanced patients, exceeding 70 % [3]. Therefore, early detection of OSCC is indispensable for improving prognosis.

Oral leukoplakia is a premalignant lesion of the oral mucosa that is characterized by a circumscribed thickening of the mucosa covered by whitish patches [4]. Although hospital-based follow-up studies have shown that between <1 % and 18 % of oral premalignant lesions will develop into oral cancer, a certain clinical subtype of leukoplakia with epithelial dysplasia has been shown to be at an increased risk for malignant transformation [5]. However, histological assessment of epithelial dysplasia has also demonstrated that not all lesions that show dysplasia will develop into oral cancer, and some will even regress [5]. Therefore, the development of other methods for predicting the malignant potential of premalignant lesions has been proposed. Recent studies have examined the molecular profiles of oral premalignant lesions in terms of the risk for malignant transformation [6]. Genetic alterations and molecular abnormalities have been identified in oral premalignant lesions. A loss of heterozygosity (LOH) at chromosome 9p and 3p and the absence of p19, a tumor-suppressor protein, are frequently observed in oral premalignant lesions [7, 8].

Although genetic alterations in epithelial cells are essential for the development of premalignant lesions, recent studies have shown that the nature of the tumor microenvironment and circumjacent stromal cells, including infiltrated immune cells, can significantly modify the outcome of these alterations [9, 10]. Numerous studies have demonstrated that tumor-associated macrophages (TAMs) initiate and promote tumorigenesis in many types of solid tumors [11–13], and a strong correlation between an abundance of TAMs and poor prognosis has been demonstrated in breast, prostate, cervical, and bladder cancers [11]. However, contrary to their tumor promoting function, TAMs that infiltrated colon and lung cancers have been associated with a better prognosis in patients [14–18]. Analysis of the phenotypes of the infiltrated TAMs revealed that the TAMs involved in poor patient prognosis share many common features with alternatively activated macrophages or M2 macrophages, which express high levels of the scavenger receptors CD163 and CD204, high levels of the chemokines CCL17, CCL22 and CCL24, and low levels of IL-12 [12, 19]. In contrast to alternatively activated macrophages, the TAMs associated with a better patient prognosis share a phenotype with classically activated macrophages or M1 macrophages, which express HLA-DR, inducible nitric oxide synthase (iNOS), and tumor necrosis factor-α (TNF-α) [17, 18]. These lines of evidence indicate that the functional competence of macrophages is heterogeneous and that the functional properties are acquired and modified in response to changes in the tumor microenvironment [12, 13].

Previous studies have observed the increased infiltration of mononuclear cells in oral premalignant lesions and OSCC [20–24]. We and others have previously observed an increased number of TAMs during the progression of OSCC, and this number positively correlates with the histopathological grade of OSCC and poor prognosis in OSCC patients [25–29]. These results suggest that TAMs participate in the progression and development of OSCC. Although the phenotypes of TAMs in various types of solid tumors have been extensively characterized, the phenotypes and functional properties of the TAMs that infiltrate premalignant lesions of solid tumors remain to be determined. This study aimed to examine TAM density in oral leukoplakia, a premalignant lesion of the oral cavity, and to characterize the macrophage phenotype (M1 or M2). We also investigated the involvement of infiltrated T cells that contribute to the induction of macrophage phenotypes.

## Methods

### Tissue specimens

Biopsy specimens were obtained from patients treated at the Division of Oral and Maxillofacial Surgery, Meikai University School of Dentistry. A total of 30 specimens diagnosed as oral leukoplakia were used in this study, and the clinicopathological characteristics of the patients examined in this study are listed in Table 1. Median age at the time of diagnosis was 61.0 years old, and 20 of the 30 patients were men. The most frequently affected site was the gingiva (43.3 %), followed by the buccal mucosa (26.7 %), tongue (13.3 %), palate (10 %), and lip (6.7 %). Biopsy specimens (seven from the tongue, two from the gingiva, and one from the palate) diagnosed as normal oral mucosa were used as controls. The current study was reviewed and approved by the Research Ethics Committee of Meikai University School of Dentistry (reference #: A0290), and written informed consent for participation in this study was obtained from the patients. These tissues were fixed for 24–48 h in 4 % formaldehyde freshly prepared from paraformaldehyde in phosphate-buffered saline (PBS) at 4 °C. The tissue specimens were sliced into 4-μm sections and mounted onto 3-aminopropyltriethoxysilane-coated glass slides. Three oral pathologists independently examined the hematoxylin and eosin slides and re-evaluated the histological diagnosis based on the WHO Classification of Head and Neck Tumors [30]. When the diagnoses were not in agreement, the biopsy specimen was re-examined and discussed until a consensus was reached.

**Table 1:** Clinicopathological characteristics of the patients with oral precancerous lesions

Clinical variables	% (n)
**Age**	
<60	33.3 (10)
≧60	66.7 (20)
Mean	61.0 years
Gender	
Male	66.7 (20)
Female	33.3 (10)
**Region**	
Gingiva	43.3 (13)
Buccal mucosa	26.7 (8)
Tongue	13.3 (4)
Palate	10.0 (3)
Lip	6.7 (2)
**Histological grade**	
Without dysplasia	16.7 (5)
Mild	23.3 (7)
Mild to moderate	16.7 (5)
Moderate	16.7 (5)
Moderate to severe	13.3 (4)
Severe	13.3 (4)

### Immunohistochemical staining

The tissue sections were deparaffinized, immersed in 10 mM citrate buffer (pH 6.0) and heated in a microwave oven for 15 min for antigen retrieval. For CD68 antigen retrieval, the tissue sections were treated with proteinase K (20 μg/ml, Roche Diagnostics, Basel, Switzerland) at room temperature for 15 min. After rinsing in PBS, the sections were incubated with 3 % hydrogen peroxide in methanol for 10 min to block endogenous peroxidase activity. Endogenous avidin and biotin were blocked using the Avidin/Biotin Blocking Kit (Zymed Laboratories, San Francisco, CA, USA) at room temperature for 10 min. To reduce nonspecific antibody binding, the samples were exposed to 2 % bovine serum albumin (BSA) for 30 min. A list of the primary antibodies used in this study is shown in Table 2. Tissue sections were incubated with primary antibody at 4 °C in a humidified chamber overnight. Then, the tissue sections were washed in PBS and incubated with horseradish peroxidase-labeled anti-mouse or anti-rabbit antibodies (Dako EnVision System, HRP-Labeled Polymer, Dako, Kyoto, Japan) for 30 min. Peroxidase activity was visualized by immersion of the tissue sections using the AEC Substrate Kit (Dako), which produced a brown reaction product. Finally, the tissue sections were counterstained with Mayer’s hematoxylin and mounted. As a negative control, the primary antibody was replaced with 2 % BSA.

**Table 2:** Primary antibodies used in this study

Antigen	Marker	Antibody	Dilution	Vendor
CD68	Pan-MΦ	Mouse anti-hCD68 Mab	1:80	Dako, Glostrup, Denmark
CD80	M1 MΦ	Mouse anti-hCD80 Mab	1:200	R&D systems, Minneapolis, MN, USA
CD163	M2 MΦ	Mouse anti-hCD163 Mab	1:200	Leica, Wetzlar, Germany
CD4	Th	Mouse anti-hCD4 Mab	1:100	NordiQC organization, Aalborg Denmark
CD8	CTL	Mouse anti-hCD8 Mab	1:100	Dako, Glostrup, Denmark
CCR4	Th2	Mouse anti-hCCR4 Mab	1:100	Novus biological, Littleton, CO, USA
CCR5	Th1	Rabbit anti-hCCR5 pab	1:200	Abcam, Cambridge, UK
CXCR3	Th1	Mouse anti-hCXCR3 Mab	1:200	R&D systems, Minneapolis, MN, USA
CXCL9	Chemokine Th1	Mouse anti-hCXCL9 Mab	1:200	R&D systems, Minneapolis, MN, USA
STAT1	IFNγ-inducible gene	Rabbit anti-hSTAT1 Pab	1:200	Santa Cruz, Santa Cruz, CA, USA
Phospho-STAT1	pSTAT1 (Tyr701)	Rabbit anti-pSTAT1 Mab	1:50	Cell signaling, Boston, MA, USA

*CD* cluster of differentiation, *Mab* monoclonal antibody, *Pab* polyclonal antibody, *Th* helper T lymphocyte, *CTL* cytotoxic T lymphocyte, *CCR4 CC* chemokine receptor 4, *CXCR3* CXC chemokine receptor 3, *CXCL9* CXC chemokine ligand 9, *STAT1* signal transducer and activator of transcription

To evaluate the positively stained cells after incubation with each antibody, three high-power magnification fields (200×) with the most abundant distribution of positive cells were selected from each specimen. The numbers of positively stained and unstained cells were counted. The data are expressed as the mean percentage of the ratio of the number of positive cells relative to the total number of cells.

### Double-labeled fluorescent immunostaining

The tissue sections were deparaffinized and immersed in Tris–HCl buffered saline (TBS: pH 7.4) supplemented with 0.5 % Triton-X 100 (Bio-Rad Laboratories, Hercules, CA, USA) and 3 % H_2_O_2_ at room temperature for 60 min. All sections were pre-blocked in 10 % non-immune goat serum (Zymed) for 1 h at room temperature to reduce nonspecific antibody binding. After being rinsed in PBS, the tissue sections were incubated with mouse anti-human CD163 monoclonal antibody (1:100 dilution) and rabbit anti-human STAT1 polyclonal antibody (1:500) or rabbit anti-phosphorylated (Try701) STAT1 monoclonal antibody (1:200) for 60 min at room temperature. The tissue sections were subsequently incubated with Alexa Fluor 488 goat anti-mouse IgG antibody at 1:2000 (Life Technologies, Carlsbad, CA, USA) and Alexa Fluor 546 goat anti-rabbit IgG antibody at 1:2000 (Life Technologies) for 60 min at room temperature. The sections were again washed in TBS prior to being cover-slipped with anti-fade mounting medium (ProLong Antifade Kit, Life Technologies). The primary antibodies were omitted in the control experiments to verify the absence of secondary antibody binding. The stained slides were viewed on a laser scanning confocal microscope (TCS SP-2, Leica, Bensheim, Germany).

### Statistical analysis

The significant differences in the numbers of positively stained cells among the various pathological grades were tested using the Kruskal-Wallis nonparametric test. Correlations between the various types of infiltrated immune cells were tested using nonparametric Spearman’s rank analysis. Two-sided *p* values of <0.05 were judged to be significant.

## Results

### Infiltration of CD163^+^ macrophages in leukoplakia

**Fig. 1:**
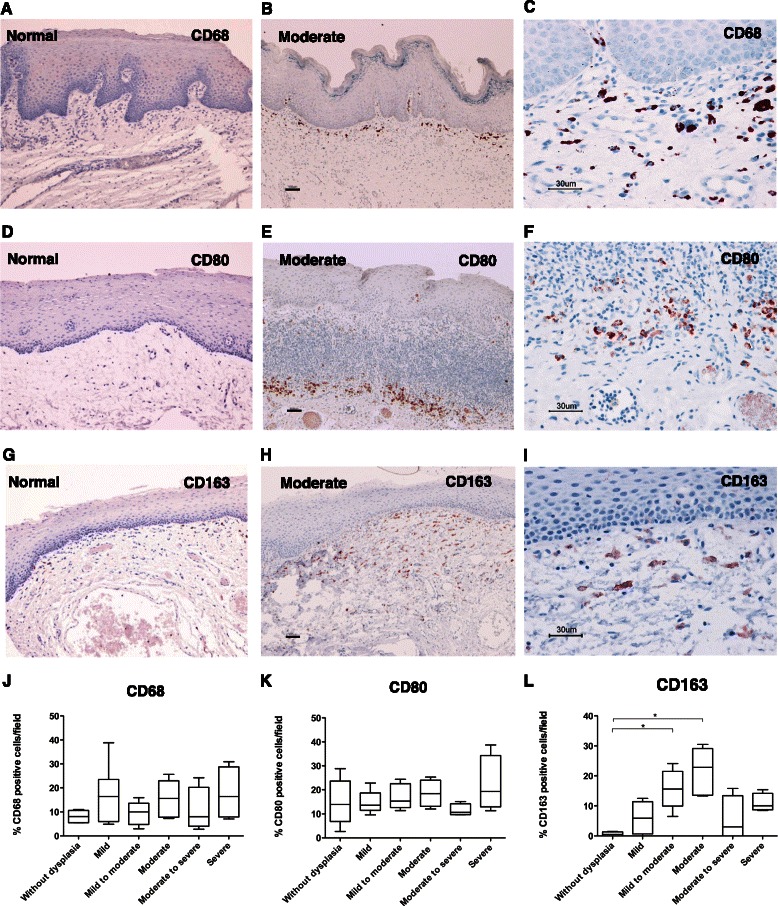
CD68^+^, CD80^+^, and CD163^+^ cells in oral normal mucosa and leukoplakia. Immunoreactivity against anti-CD68 (**a**-**c**), anti-CD80 (**d**-**f**), and anti-CD163 (**g**-**i**) in normal oral mucosa (**a**, **d**, **g**) and moderate grades (**b**, **c**, **e**, **f**, **h**, **i**) of oral leukoplakia (original magnification: A,B,D,E,G,H × 100, scale bar = 100 μm; C, F, I, ×400, scale bar = 30 μm). Percentages of CD68^+^ (**j**), CD80^+^ (**k**), and CD163^+^ (**l**) cells for various histological grades of leukoplakia are shown. Data are expressed as box plots indicating the maximum, median, and minimum values. Statistically significant differences were observed in CD163^+^ cells in the mild to moderate and moderate grades of leukoplakia compared to cases without dysplasia. (*p < 0.05, Dunn test)

We examined the infiltration of macrophages for various pathological grades of leukoplakia using antibodies to CD68 [31], CD80 [12, 32], and CD163 [33]. Few cells were positively stained for these macrophage markers in the normal mucosa (Fig. 1a, d, g), while CD68^+^, CD80^+^ and CD163^+^ cells were observed in specimens from leukoplakia lesions (×100: Fig. 1b, e, h and × 400: c, f, i). The majority of the infiltrated macrophages were distributed in the subepithelial stroma. Although the percentages of infiltrated CD68^+^ (Fig. 1j) and CD80^+^ (Fig. 1k) cells did not differ significantly by pathological grade, the number of infiltrated CD163^+^ cells was significantly increased in mild to moderate and mild dysplasia compared to samples without dysplasia (Fig. 1l).

**Fig. 2:**
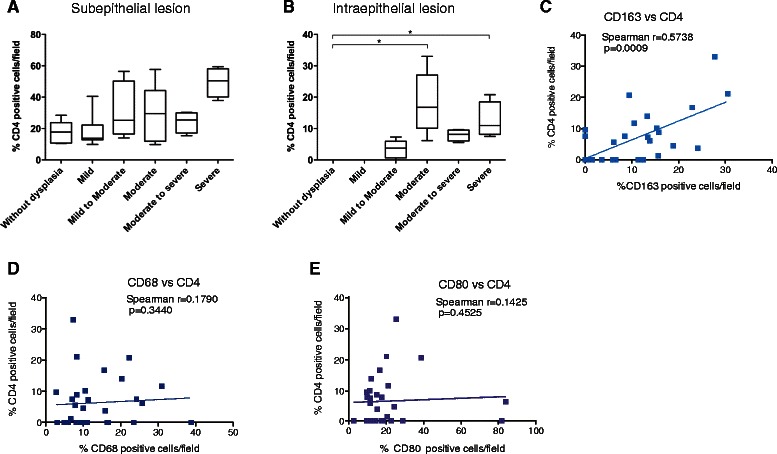
CD4^+^ T cells in subepithelial and intraepithelial lesions of various histological grades of oral leukoplakia. Percentages of CD4^+^ T cells in subepithelial (**a**) and intraepithelial (**b**) lesions of various histological grades of leukoplakia are shown. Data are expressed as box plots indicating the maximum, median, and minimum values. Statistically significant differences were observed in intraepithelial lesions of the moderate and severe grades of dysplasia compared to cases without dysplasia (*p < 0.05, Dunn test). Correlation between infiltrated CD163^+^ and CD4^+^ cells in leukoplakia (**c**). Statistically significant differences were determined using Spearman’s rank correlation coefficient analysis (*p* = 0.0009). There were no statistically significant correlations between CD68^+^ and CD4^+^ cells (**d**) and CD80^+^ and CD4^+^ cells (**e**)

Because the tumor microenvironment modulates the functional properties of TAMs, we examined the infiltration of T cells in leukoplakia. Both CD4^+^ (Fig. 2) and CD8^+^ (data not shown) T cells were observed in the subepithelial stroma of leukoplakia. Interestingly, a significant increase in the percentages of CD4^+^ T cells was detected in the intraepithelial lesions of moderate and severe dysplasia (Fig. 2b). Spearman’s rank correlation coefficient was used to assess the potential relationship between CD163^+^ macrophages and CD4^+^ T cells. The percentages of CD163^+^ macrophages positively correlated with the intraepithelial CD4^+^ T cells (*p* < 0.0009; Fig. 2c). However, there were no significant correlations between CD68^+^ cells and CD4^+^ T cells, nor between CD80^+^ cells and CD4^+^ T cells (Fig. 2d, e).

### Infiltration of CXCR3^+^ T cells in leukoplakia and correlation with STAT1^+^ cells

**Fig. 3:**
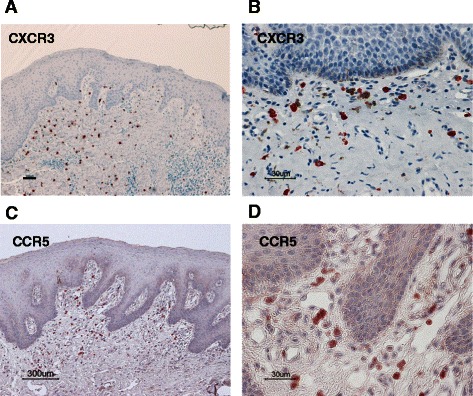
Immunohistochemical staining of oral leukoplakia with the anti-CXCR3 and anti-CCR5 antibodies. Immunoreactivity against anti-CXCR3 (**a**, **b**) and anti-CCR5 (**c**, **d**) antibodies for moderate grades of oral leukoplakia (original magnification: A, C: ×100; B, D: ×400). CXCR3^+^ and CCR5^+^ Th1 cells were mainly distributed in the subepithelial lesion. Scale bar = 100 μm (**a**), 300 μm (**c**), and 30 μm (**b**, **d**)

Th1-derived IFN reportedly induces classically activated M1 macrophages, whereas Th2-derived IL-4 and IL-13 induce alternatively activated M2 macrophages [12, 19]. To further analyze the subset of infiltrated T cells that affect the phenotype of TAMs, we immunohistochemically examined the infiltrated CD4^+^ T cells using antibodies to chemokine receptor CXCR3 and CCR5, markers for Th1 cells, and antibodies to CCR4, a marker for Th2 cells [34]. Although CCR4^+^ T cells were rare in normal mucosa and in leukoplakia lesions (data not shown), CXCR3^+^ (Fig. 3a, b) and CCR5^+^ (Fig. 3c, d) T cells abundantly infiltrated the subepithelial stroma of leukoplakia. These results indicate that CD4^+^ Th1 cells are the predominant subset of T cells that infiltrate leukoplakia.

**Fig. 4:**
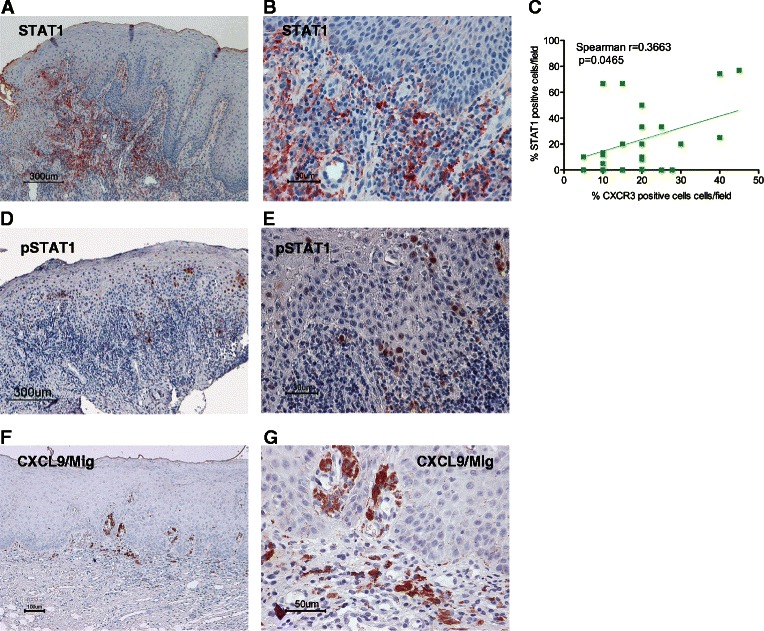
Immunohistochemical staining of oral leukoplakia with the anti-STAT1, anti-pSTAT1 and anti-CXCL9/Mig antibodies Immunoreactivity against anti-STAT1 (**a**, **b**), anti-tyrosine (Try701)-phosphorylated- STAT1 (pSTAT1) (**d**, **e**) and anti-CXCL9/Mig (**f**, **g**) antibodies for moderate grades of oral leukoplakia (original magnification: A, D, F: ×100; B, E, G: ×400). STAT1^+^ and CXCL9^+^ cells were mainly distributed in the subepithelial lesion. Scale bar = 300 μm (**a**, **d**), 100 μm (**f**), 50 μm (**e**, **g**) and 30 μm (**b**). Correlation between infiltrated CXCR3^+^ and STAT1^+^ cells in leukoplakia (**c**). Statistically significant differences were determined using Spearman’s rank correlation coefficient analysis (*p* = 0.0465)

Because Th1 cells produce IFN, which induces M1 macrophages, we next assessed whether the IFN-inducible gene products STAT1 [35] and CXCL9/Mig, a chemokine for Th1 [36], were expressed in leukoplakia (Fig. 4). STAT1^+^ cells were widely distributed in the subepithelial lesions of leukoplakia. The percentages of CXCR3^+^ cells positively correlated with the percentages of STAT1^+^ cells (*p* = 0.0465; Fig. 4c). Tyrosine-phosphorylated STAT1 (pSTAT1), an active form of STAT1, was also detected in the lesions (Fig. 4d, e), though the frequency of pSTAT1-positive cells was lower than that of STAT1-positive cells. Cells positive for the IFN-inducible chemokine CXCL9 were also observed in the subepithelial lesion of leukoplakia (Fig. 4f, g). Taken together, these results indicate that the leukoplakia lesions form a Th1-dominated microenvironment and suggest that Th1-derived IFN affects the infiltrated macrophages to polarize the M1 phenotype.

### Colocalization of CD163^+^ cells with STAT1 in leukoplakia

**Fig. 5:**
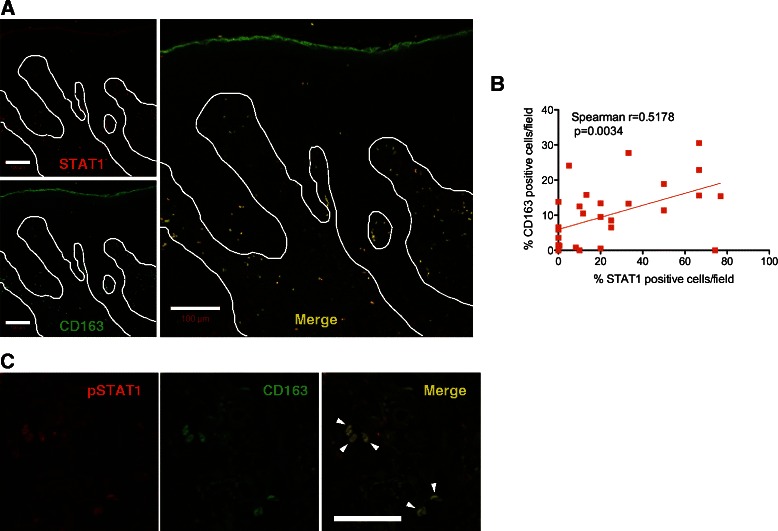
Colocalization of CD163^+^ TAMS with STAT1 in oral leukoplakia. Double-labeled fluorescent immunostaining for CD163 (*green*) and STAT1 or pSTAT1 (*red*) in oral leukoplakia with moderate dysplasia. The cells double-stained for both the anti-CD163 and anti-STAT1 antibodies are shown (**a**, *yellow*). These cells colocalized to the subepithelial lesion (original magnification: ×200). Scale bar = 100 μm. **b** Correlation between infiltrated STAT1^+^ and CD163^+^ cells in oral leukoplakia. Statistically significant differences were determined using Spearman’s rank correlation coefficient analysis (*p* = 0.0034). **c** The cells double-stained for both the anti-CD163 and anti-pSTAT1 antibodies are shown (*arrow heads*)

To further characterize the CD163^+^ macrophages in leukoplakia, we examined the coexpression of CD163 and STAT1 or pSTAT1 using double-labeling immunofluorescence (Fig. 5). CD163^+^ macrophages were distributed in the subepithelial lesion, and the majority of CD163^+^ cells located in the papillary dermis colocalized with STAT1 (Fig. 5a). The percentages of CD163^+^ macrophages and STAT1^+^ cells were positively correlated (*p* = 0.0034; Fig. 5b). Although the percentages of single-stained cells for CD163 and STAT1 were 16.4 % and 32.1 %, respectively, the percentage of double-stained cells was 51.5 % (*n* = 4). The CD163^+^ cells also coexpressed pSTAT1 (Fig. 5c). These results indicate that CD163^+^ macrophages in oral leukoplakia coexpress active STAT1 and suggest that the CD163^+^ macrophages possess an M1 phenotype in a Th1-dominated microenvironment.

## Discussion

Macrophages are one of the major cellular components in the tumor microenvironment, and macrophages have been considered to be crucial to tumor development [13, 37]. Although a number of studies have reported the phenotypes and properties of these macrophages (i.e., TAMs) in various human solid tumors, including oral squamous cell carcinoma, the phenotypes of TAMs in the premalignant lesions of these solid tumors remain to be determined. Specifically, changes in the phenotypes and functional properties of TAMs in premalignant lesions during tumor development have not yet been characterized. To this end, we examined dissected specimens from 30 patients who underwent biopsy and received a diagnosis of oral leukoplakia, an oral premalignant lesion, by immunohistochemical analysis of several macrophage and T cell markers. The results demonstrated that although CD163 has been considered a M2 macrophage marker in many solid tumors [38–41], the CD163^+^ macrophages in oral leukoplakia appear to possess an M1 phenotype characterized by the expression of IFN-inducible gene products. Furthermore, infiltrated CXCR3^+^ and CCR5^+^ Th1 cells, a major IFN-producing cell type, were also observed in the tumor microenvironment. These results suggest that the infiltrated Th1 cells, which produce IFN, affect the phenotype of CD163^+^ macrophages in oral premalignant lesions.

The macrophages were classified as M1 (classically activated) and M2 (alternatively activated) macrophages based on the expression of macrophage gene products, including receptors, cytokines, and effector molecules, induced by classical macrophage-activating stimuli such as Th1-derived IFN or the Th2-derived anti-inflammatory cytokines IL-4 and IL-13 [12, 19, 42]. M1 macrophages produce large amounts of pro-inflammatory cytokines, reactive oxygen intermediates and reactive nitrogen intermediates, such as nitric oxide (NO), which contribute to the anti-tumor activity of macrophages [12]. In contrast, M2 macrophages have been suggested to contribute to angiogenesis, tissue remodeling, and tumor progression by inducing the expression of mannose receptors, scavenging receptors, angiogenic factor such as vascular endothelial growth factor (VEGF), and low levels of pro-inflammatory cytokines [12]. Although the M1/M2 concept of macrophage polarization helps explain the functional properties of macrophages in various infectious and immunological diseases, recent accumulated evidence has shown that the infiltrated TAMs in human solid tumors appear to consist of a heterogeneous population [40, 43]. TAMs in human cutaneous SCC appear to consist of a mixed subpopulation of CD163^+^ cells that express M1 markers, M2 markers or both M1 and M2 markers [43]. In agreement with this previous study, we have also demonstrated that CD163^+^ cells express an M1 marker in oral premalignant lesions. Related to these findings, we and others have previously reported that CD163^+^ macrophages are the major TAMs in OSCC and that an increased number of CD163^+^ macrophages correlates with a poor prognosis [27, 28, 44, 45]. Our preliminary double-labeling immunofluorescence data for CD163 and STAT1 shows that the CD163^+^ TAMs in OSCC also coexpress STAT1. These results suggest that CD163^+^ TAMs with the M1 phenotype persist in the tumor microenvironment from the premalignant to the malignant stage. The functional heterogeneity of the CD163^+^ TAMs that express M1 markers in OSCC in terms of antitumor or protumor potency remains to be determined. CD163^+^ TAMs need to be further characterized to better understand the role of TAMs in the progression of OSCC.

TAMs acquire functional competence in response to various cytokines and mediators encountered within the tumor microenvironment [46]. Tumor-associated immune cells, as well as tumor cells themselves, are the major sources of mediators that affect the functional properties of TAMs. A mouse tumor model of mammary carcinomas demonstrated that IL-4-expressing CD4^+^ T cells indirectly promote the invasion and metastasis of mammary adenocarcinomas by promoting the protumor function of TAMs [47]. The importance of Th2 cytokines, including IL-4, IL-10, and IL-13, in the regulation of the protumor functions of TAMs has also been demonstrated in human lung adenocarcinomas [40, 48]. However, the role of infiltrated T cells in the polarization of TAMs in premalignant lesions of human solid tumors is not completely understood. In the present study, we evaluated the relationship between infiltrated T cells and the polarization of TAMs in oral leukoplakia and found a positive correlation between the numbers of CD4^+^ T cells and CD163^+^ macrophages. Intriguingly, the infiltrated CD4^+^ T cells in oral leukoplakia consisted of CXCR3^+^ and CCR5^+^ Th1 cells, a major IFN-producing cell type, and CCR4^+^ Th2 cells were rare in the lesion. Consistent with the increased infiltration of Th1 cells and STAT1^+^ cells, the expression of an IFN-inducible gene product [35] was also increased in the lesion. These results suggest that the tumor microenvironment of oral leukoplakia creates a Th1-dominated microenvironment that polarizes TAMs toward the M1 phenotype. Our double-labeled immunofluorescence analysis demonstrated that CD163^+^ macrophages coexpressed active STAT1 (pSTAT1). Thus, it is highly likely that the infiltrated Th1 cells modulate the phenotype of TAMs in oral premalignant lesions.

The recruitment of CXCR3^+^ Th1 cells is mediated by IFN-inducible chemokines such as CXCL9 and CXCL10 [49], which are produced by a variety of cell types, including epithelial cells, fibroblasts, and macrophages, in response to IFNs [50, 51]. IFNs and TNF or CD40 ligand synergistically induce the expression of these chemokines [52, 53]. Although the initial triggering molecules that induce these chemokines are unknown, epithelial cells in the dysplastic lesion, which are continuously stimulated by carcinogens and are genetically altered, may produce these chemokines. In fact, our immunohistochemical analysis demonstrated that CXCL9 was present in the subepithelial lesion of leukoplakia. After the recruitment of CXCR3^+^ Th1 cells, the secretion of IFN could further skew the oral premalignant lesions toward a Th1-dominated microenvironment. Intriguingly, a previous proteomic analysis of OSCC revealed that the IFN signaling pathway is significantly enhanced in OSCC lesions and that the expression of IFN-inducible gene products, including STAT1, was up-regulated [54]. Taken together, these results suggest that the persistent IFN-stimulating environment from the premalignant to malignant lesion may allow tumor cells to acquire resistance to the antitumor responses of IFNs via cancer immunoediting [55]. IFN-induced M1 macrophages have been shown to act as important effectors during cancer immunoediting in a mouse tumor model [56]. Further *in vivo* studies using animal models of OSCC are needed to explore the functional role of IFN-stimulated M1 macrophages in the progression of malignant transformation.

We histopathologically graded the biopsy specimens of oral leukoplakia based on the WHO classification [30] and explored the relationship between the histological grading and the levels of infiltrated immune cells. Significant increases in CD163^+^ macrophages (Fig. 1) and intraepithelial CD4^+^ T cells (Fig. 2b) were observed in moderate dysplasia compared to samples without dysplasia (Fig. 1). The immunohistochemical analysis showed that CD163^+^ macrophages were mainly distributed in the subepithelial region. Although the etiological roles of CD163^+^ macrophages and intraepithelial CD4^+^ T cells in the development of dysplasia are unclear, CD163^+^ macrophages may contribute to the infiltration of intraepithelial CD4^+^ T cells. Because intraepithelial lymphocyte migration is accompanied by fragmentation of the basement membrane [57], CD163^+^ macrophages may secrete matrix metalloproteinases (MMPs) that degrade the basement membrane [58]. A loss of basement membrane components has been correlated with the invasive potential of malignant epithelial neoplasms [59]. CD163^+^ macrophages and the infiltration of T cells into the epithelial lesion may contribute to the early architectural disturbance of the epithelium during the development of dysplasia.

## Conclusion

In summary, we have identified an increase in CD163^+^ macrophages in oral premalignant lesions and shown that CD163^+^ macrophages coexpress STAT1, an M1-related marker. Our results also suggest that recruited CXCR3^+^ and CCR5^+^ Th1 cells that produce IFN in the dysplastic lesion influence the polarization of the TAMs toward an M1 phenotype.

## Abbreviations

TAMs: Tumor-associated macrophages
Th: helper T cells
CD: Cluster of differentiation
CXCR3: Chemokine (C-X-C motif) receptor 3
CCR4: Chemokine (C-C motif) receptor 4
CXCL9: Chemokine (C-X-C motif) ligand 9
STAT1: Signal transducer and activator of transcription
IFN: Interferon-
SCC: Squamous cell carcinoma
OSCC: Oral squamous cell carcinoma
CCL: Chemokine (C-C motif) ligand
IL: Interleukin
TNF-: tumor necrosis factor-
M1: Macrophage phenotype 1
M2: macrophage phenotype 2
BSA: Bovine serum albumin
PBS: Phosphate-buffered saline
TBS: Tris–HCl-buffered saline
NO: Nitric oxide
VEGF: Vascular endothelial growth factor
